# Gene models of four *Schistocephalus solidus* aminoacyl-tRNA synthetases

**DOI:** 10.17912/micropub.biology.002355

**Published:** 2026-09-16

**Authors:** Julian Flanagan, Amanda K. Hund, Joseph Chihade

**Affiliations:** 1 Carleton College, Northfield, MN, United States; 2 Department of Biology, Carleton College, Northfield, MN, United States; 3 Department of Chemistry, Carleton College, Northfield, MN, United States

## Abstract

Aminoacyl-tRNA synthetases (ARSs) are the enzymes responsible for pairing tRNAs with amino acids during protein synthesis. Despite their conserved function, important features of these enzymes vary between species. Because previous research on animal ARSs has predominantly focused on human enzymes, relatively little is known about ARSs from non-vertebrate parasites. To examine helminth-specific ARS features, we used available genomic and RNA-seq data to produce corrected gene models of four ARSs from *Schistocephalus solidus* , a cestode parasite. Predicted gene models were verified and extended by PCR amplification and sequencing of *S. solidus* cDNA, revealing single nucleotide variations we attribute to geographical variation.

**Figure 1. S. solidus ARS genes and strategies for cDNA amplification:** A) Aminoacyl-tRNA synthetases catalyze specific tRNA charging required for protein synthesis. B) *Schistocephalus solidus* removed from the abdominal cavities of threespine stickleback fish. C-F) Summary of *S. solidus* ARS genes and strategies for cDNA amplification. Existing gene models are displayed in blue. Additional portions identified by examination of RNAseq data or by amplification of unknown sequences through 3' or 5' RACE are indicated by white rectangles. Unsequenced (N in genomic sequence) regions are shown in gray. Where possible, sequencing scaffolds (ovals, SSLN_scaffold *x* ), annotated gene IDs (SSLN_000 *x* ), and descriptions from BioProject PRJEB527 are noted. Arrows indicate relative positions of PCR primers used during cDNA amplification. G) Representative example of revisions made to *S. solidus* gene models. This data taken from the MARS 5' region. Existing coding sequence annotation is shown in light blue, RNAseq dataset SRR2966897 coverage in dark blue, and the revised model in pink. Colored boxes represent exons and thin lines represent introns. Corrections to the gene model include addition of missing exons (left), removal of exon unsupported by RNAseq (center) and adjusted exon boundaries (right).

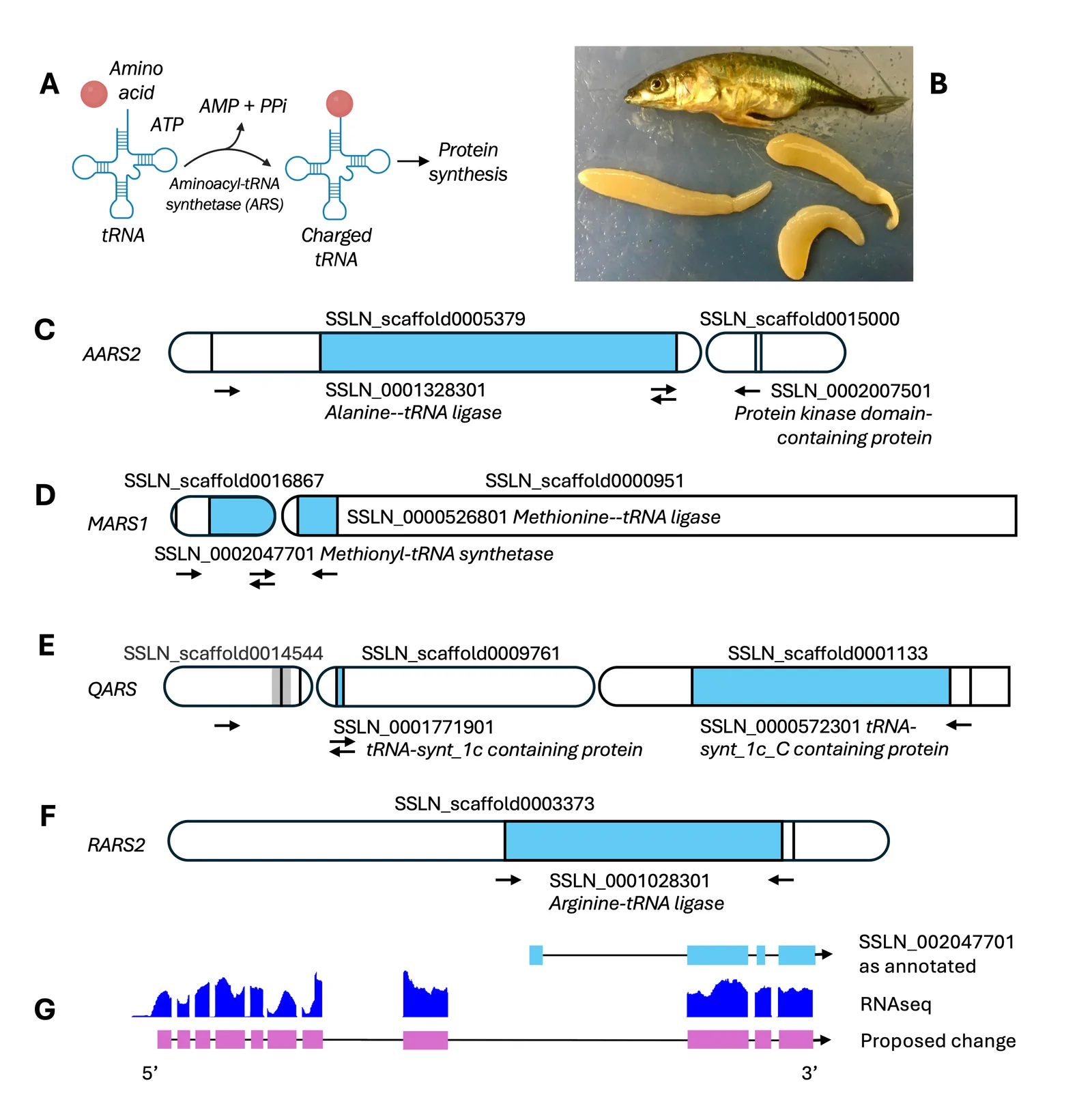



## Description

Aminoacyl-tRNA synthetases (ARSs) catalyze the two-step reaction leading to formation of an ester linkage between an amino acid and the 3' end of a corresponding tRNA, an essential and universal step in protein synthesis ( Fig. 1A ). While the core catalytic mechanism is conserved among enzymes with the same amino acid specificity, secondary features of eukaryotic ARSs provide opportunities for inter-species variation (Pham et al., 2014; Tennakoon and Cui, 2024).

Among metazoan species, these differences are most obvious in two groups of ARSs. The first includes a number of cytosolic ARSs that assemble, together with accessory proteins, to form the multisynthetase complex (MSC), which carries out a variety of non-translational functions. ARSs that are part of the complex often have appended domains, which facilitate interactions with other MSC proteins and can differ considerably between phylogenetic classes (Gomez and Ibba, 2020; Kim and Kang, 2022) . The second includes mitochondrial ARSs, which are translated in the cytosol and transported to the mitochondria, where they recognize mitochondrially-encoded tRNAs. These mitochondrial tRNAs frequently deviate from classical tRNA structure and specific structural features vary between organisms, particularly among bilaterian animals (Helm et al., 2000). Perhaps because of this variation in their substrates, mitochondrial ARSs also exhibit class-specific features, particularly in tRNA binding domains (Kuhle et al., 2020).

*Schistocephalus solidus* is a tapeworm parasite of threespine stickleback fish, birds, and freshwater copepod invertebrates ( Fig. 1B ). *S. solidus* is an established model organism for studying host-parasite interactions and methods have been developed for laboratory breeding (Orr and Hopkins, 1969) *.* Two *S. solidus* genome projects have been published, using samples from northern Germany (BioProject PRJEB527 ) (Coghlan et al., 2018) and Quebec, Canada (BioProject PRJNA576252 ) (Berger et al., 2021), the first of which includes an available annotation. These resources make *S. solidus* an attractive system in which to examine cestode-specific features of ARSs.

In this work, we established the protein coding regions of four ARS genes, encoding two potential components of the cytosolic MSC (cytosolic methionyl-tRNA synthetase, MARS1, and glutaminyl-tRNA synthetase, QARS) and two mitochondrial (mt) enzymes (mt alanyl-tRNA synthetase, AARS2, and mt arginyl-tRNA synthetase, RARS2). We initially accessed gene models from the 2018 annotation *via* WormBase ParaSite (Howe et al., 2017). Keyword searches yielded predicted coding sequences that are incomplete, lacking expected features of functional ARS enzymes. Using TBLASTN to search the same genomic sequence, using homologs *Taenia solium* , *Echinococcus multilocularis* , and *Echinococcus granulosus* as queries and for reference, revealed both additional homologous coding sequences and unannotated regions. RARS2 coding sequences are limited to a single scaffold ( Fig. 1F ), but sequences corresponding to other ARSs were found in two (AARS2 and MARS1) or three (QARS), suggesting that the corresponding genes are split across multiple scaffolds ( Fig. 1C- E). Available gene models also appeared to contain errors in predicted intron and exon boundaries. Inspired by the Genomics Education Partnership (Rele et al., 2023), we used the mapping of RNA-seq data to the 2018 genome accessible through the Ensembl Genome Browser in WormBase ParaSite to assemble revised gene models. Changes are proposed in all four gene models, with unlikely exon boundaries and missing exons most common at the beginnings and ends of predicted genes. For example, in MARS1, the seven most N-terminal exons in our model are missing from the published annotation while the most N-terminal annotated exon is not supported by RNA-seq ( Fig. 1G ).

To confirm the accuracy of our gene models and to support further *in vitro* work, we designed primers for PCR amplification of cDNA generated from *S. solidus* tissue samples collected from Lake Vancouver, Canada. 5'- (QARS) or 3'- (AARS2) RACE techniques were used in cases where the sequences of the corresponding termini could not be deduced from the genomic sequence and overlap-extension methods were used when predicted sequences spanned multiple scaffolds. PCR products were Sanger sequenced, subsequently cloned into pUC19, and resequenced using whole-plasmid methods. Results were consistent with the intron/exon boundaries in our predictions. Each coding sequence maps to a single contig in the 2021 genome, with intron/exon boundaries identical to our proposed models. Models were additionally confirmed by mapping to assembled transcriptomes generated from two distinct RNA-seq data sets.

Interestingly, a number of single-nucleotide variations were present in the coding sequences for each gene. Vancouver Island *S.solidus* cDNA sequences differed in two (QARS and RARS2) to nineteen (AARS2) positions from genomic and transcriptomic data derived from samples collected in Europe and Quebec. The majority of the 26 variations are either silent (14) or result in conservative amino acid substitutions (11). Significant genetic variation at the population level has been observed previously in *S. solidus* , even in samples obtained from relatively proximate locations (Sprehn et al., 2015; Hébert et al., 2016), and is likely to account for the differences we observed.

## Methods

*Gene model construction:*

A keyword search was performed on assembly GCA_900618435.1 in the WormBase ParaSite database (Howe et al., 2017) to locate *S. solidus* ARS genes. Predicted intron-exon boundaries were adjusted manually in accordance with RNA-seq data accessed via the WormBase ParaSite database and visual inspection of potential splice sites. RNA-seq projects utilized are also accessible via the NCBI BioProject database under accession numbers PRJNA358581 and PRJNA304161 . MAFFT (Katoh and Standley, 2013) alignments with corresponding *Taenia solium* , *Echinococcus multilocularis* , and *Echinococcus granulosus* protein sequences, obtained from Wormbase ParaSite, were used to corroborate these changes and to identify instances where genes of interest were fragmented across multiple contigs.

*cDNA preparation and amplification:*

Plerocercoid *S. solidus* specimens were collected from infected threespine stickleback at Merrill Lake in Vancouver Island, Canada (50.0614 N, -125.5628 W) during June and July of 2023. Stickleback were euthanized with a lethal dose of MS-222 and dissected in the field. Collection was performed under the British Columbia Fish Collection permit NA23-787881 and IACUC permit A21-025 through the University of Connecticut. *S. solidus* were extracted from dissected stickleback, preserved in RNA *later* (Invitrogen), then stored at -80 ºC. Approximately 30 mg of thawed frozen *S. solidus* tissue was homogenized with a microfuge pestle followed by sonication. Total RNA was extracted from homogenate using the PureLink™ RNA Mini Kit (Invitrogen). 1 µg of isolated RNA was used as a template for reverse transcription using the LunaScript® RT SuperMix Kit (NEB) protocol.

PCR primers for amplification of *S. solidus* ARS sequences were designed corresponding to the furthest 5' or 3' portions identified during this genomic analysis. For the AARS2 sequence, a T25V anchored primer was used to amplify the unknown 3' portion. Similarly, amplification of the unknown 5' portion of QARS1 cDNA was accomplished using 5' RACE with NEB Template Switching RT Enzyme Mix. When known and unknown portions were amplified as separate fragments, overlap extension PCR was used to assemble the complete gene sequence. After Sanger sequencing of PCR products established the location of initiation and termination codons, restriction sites were introduced *via* PCR to the 5' and 3' ends of each cDNA sequence and the resulting products were cloned into pUC19. Resulting plasmids were verified by whole-plasmid and Sanger sequencing.

*Gene model validation and comparison:*

Transcriptomes were assembled from RNA-Seq datasets with accession numbers SRR2966892 and SRR19629899 using rnaSPAdes (Bushmanova et al., 2019) software with default parameters. Minimap2 (Li, 2018) was used to align cDNA sequences to genomic and transcriptomic datasets.

## Data Availability

Description: Predicted amino acid and coding sequences for S. solidus ARS and gene models corresponding to each of the published genomes. Resource Type: Dataset. DOI: https://doi.org/10.22002/hv4eg-wg213
